# Electrochemical microfluidic biosensor for the detection of CD4^+^ T cells

**DOI:** 10.1038/s41378-025-00893-8

**Published:** 2025-04-09

**Authors:** Katarzyna Białas, Hui Min Tay, Chayakorn Petchakup, Razieh Salimian, Stephen G. Ward, Mark A. Lindsay, Han Wei Hou, Pedro Estrela

**Affiliations:** 1 Centre for Bioengineering & Biomedical Technologies (CBio), University of Bath, Calverton Down, Bath, BA2 7AY United Kingdom; 2 Department of Electronic & Electrical Engineering, University of Bath, Claverton Down, Bath, BA2 7AY United Kingdom; 3 School of Mechanical and Aerospace Engineering, Nanyang Technological University, 50 Nanyang Avenue, Singapore, 639798 Singapore; 4 Centre for Therapeutic Innovation, University of Bath, Claverton Down, Bath, BA2 7AY United Kingdom; 5 Department of Life Sciences, University of Bath, Claverton Down, Bath, BA2 7AY United Kingdom

**Keywords:** Electrical and electronic engineering, Engineering

## Abstract

Since the onset of the HIV epidemic, assessing CD4^+^ T-cells has become a routine procedure for evaluating immune deficiency, with flow cytometry established as the gold standard. Over time, various strategies and platforms have been introduced to improve CD4^+^ cell enumeration, aiming to enhance the performance of diagnostic devices and bring the service closer to patients. These advancements are particularly critical for low-resource settings and point-of-care applications, where the excellent performance of flow cytometry is hindered by its unsuitability in such environments. This work presents an innovative electrochemical microfluidic device that, with further development, could be applied for HIV management in low resource settings. The setup integrates an electrochemical sensor within a PDMS microfluidic structure, allowing for on-chip electrode functionalization and cell detection. Using electrochemical impedance spectroscopy, the biosensor demonstrates a linear detection range from 1.25 × 10^5^ to 2 × 10^6^ cells/mL, with a detection limit of 1.41 × 10^5^ cells/mL for CD4^+^ cells isolated from blood samples, aligning with clinical ranges for both healthy and HIV^+^ patients. The biosensor shows specificity towards CD4^+^ cells with negligible response to monocytes, neutrophils, and bovine serum albumin. Its integration with a microfluidic chip for sensor fabrication and cell detection, compact size, minimal manual handling, ease of fabrication, electrochemical detection capability, and potential for multiplexing together with the detection range make the device particularly advantageous for use in low-resource settings, standing out among other devices described in the literature. This study also investigates the integration of a microfluidic Dean Flow Fractionation (DFF) chip for cell separation.

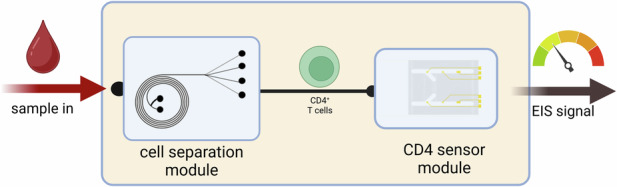

## Introduction

Human immunodeficiency virus (HIV) is a significant global health challenge, particularly prevalent across Africa, leading to immune system failure and leaving individuals vulnerable to life-threatening infections and cancers^1–3^. Limited access and high costs associated with HIV management amenities stand as one of the main challenges in low-resource settings. Many HIV-positive patients lack nearby medical facilities for essential health monitoring and treatment efficacy assessment. Diagnosis of HIV is crucial for preventing disease progression and reducing transmission rate. Access to accurate and affordable diagnostic tools, including rapid tests and laboratory-based assays, plays a pivotal role in early detection. Moreover, companion diagnostics can help monitor disease progression risk and tailor antiretroviral therapy effectively^4^. Monitoring the progression of HIV infection involves regular assessment of viral load and CD4^+^ cell count^5–7^. Viral load testing measures the amount of HIV RNA in the blood, providing insight into viral replication levels and treatment response. CD4^+^ cell count, on the other hand, reflects the status of the immune system and helps guide the initiation and adjustment of antiretroviral therapy^8^. In the initial acute stage of infection (primary infection), HIV multiplies rapidly causing a sharp drop in CD4^+^ cell count (from ~1100 cells/µL to ~500 cells/µL), which often causes flu-like symptoms. In this phase the level of HIV antigens in the blood is high. Next, in the chronic phase, the immune system tries to fight the infection and HIV-1/HIV-2 antibodies are produced. CD4^+^ cell count initially increases (to ~700 cells/µL), but usually not to the pre-infection level. Despite a patient being asymptomatic, their immune system is gradually damaged and is not capable of effectively fighting the infection. At critically low CD4^+^ cell count (below ~200 cells/µL), there is a high risk of developing opportunistic infections, which are life-threatening for an immunocompromised patient^9^. Given the critical role of CD4^+^ cell count in guiding HIV treatment and assessing immune function, reliable and accessible CD4^+^ detection is essential for managing HIV infection and disease progression. However, current CD4^+^ detection technologies are often limited by their reliance on specialized equipment, high costs, and laboratory infrastructure, making them less suitable for use in low-resource settings.

Although gold standard methods for HIV biomarker diagnosis and monitoring, such as flow cytometry, PCR, and ELISA, are highly effective^10^, they have limitations, including high sample volume requirements, the need for fluorescently labeled antibodies, sophisticated equipment, and time-consuming protocols that require trained professionals for data acquisition and analysis. To address these challenges, researchers have been focusing on developing compact, simplified, and efficient detection tools specifically tailored for deployment in resource-constrained environments, where the majority of HIV-positive patients reside^11–22^. Among these, electrochemical sensors integrated with microfluidic systems have emerged as promising platforms yielding easy to use, portable, and cost-effective devices suitable for point-of-care applications^11–23^. Furthermore, the need for precise biomarker targeting within complex biological matrices has driven interest in affinity-based biosensors, with antibodies being among the most reliable options due to their high specificity and sensitivity, which are essential for accurate detection in compact, practical formats^24–28^. Several commercially available CD4^+^ cell counting devices are on the market, including PointCareNOW^TM^, CyFlow® miniPOC, BD FACSPresto, MBio^TM^ Diagnostic CD4 System, Alere Pima^TM^ Analyser, Visitect CD4, Zyomyx CD4 Test, and Daktari^TM^ CD4^+^. However, they each have limitations, such as accuracy issues, off-instrument sample preparation, high costs, low throughput, or semi-quantitative results, making them less ideal for use in low-resource settings. Additionally, most of these devices rely on optical or fluorescence detection, which can increase equipment complexity and maintenance costs. The PointCareNOW^TM^, a flow cytometry-based device, was shown to be inaccurate in five independent evaluations^29^. The CyFlow^®^ miniPOC, although portable and accurate, requires an off-instrument sample preparation, expensive instrumentation, and trained staff for its operation and maintenance^17,30^. The BD FACSPresto’s limitations include lower sensitivity and higher misclassification rates in clinical settings, which may result in misidentifying patients who require treatment^31^. The Alere Pima™ Analyser is another fluorescence-based device; however, unlike the aforementioned devices, it operates in a static mode through image analysis. Minimal sample handling and processing, along with a sample within a disposable cartridge, reduces the risk of contamination. On the other hand, the device is considered low throughput, with a maximum of 20 tests performed per day^17^. The Visitect CD4 is a rapid colorimetric lateral flow test which provides only a semi-quantitative readout (“treat” or “no treat”). The Zyomyx CD4 Test stands out as an electronics-free device in which CD4^+^ cells bind to heavy particles, leading to complex sedimentation and a readout on a scale similar to that of a thermometer^17,32^. Finally, the Daktari™ CD4 Counter stands out as the sole device that operates on an electrochemical basis, measuring electrical impedance for CD4^+^ cell counting. However, to the best of our knowledge, independent validation is not available, and the company has discontinued the device.

Extensive research is underway to improve existing technologies for point-of-care CD4^+^ cell detection, with a particular focus on addressing the limitations of current electrochemical sensors. While electrochemical sensors offer great potential for affordability, portability, and rapid response, their development for CD4^+^ cell counting has faced several challenges. These include issues such as lengthy fabrication processes, variability in sensor response, and limited integration with microfluidic platforms, which are crucial for creating self-contained, user-friendly devices suitable for low-resource settings. The limited number of electrochemical sensors reported in the literature highlights a considerable opportunity to advance simple detection platform designs in this field^33,34^. A device proposed by Carnelli et al. performs immunomagnetic separation of target cells from whole blood using a magneto-electrode, providing a simple and reliable method for sample preconcentration and removal of potentially interfering molecules in blood^34^. However, this sensor was not miniaturized into a self-contained device. In contrast, a CD4^+^ cell sensor developed by Kim et al. was miniaturized onto a chip, but its fabrication is a lengthy process, and the sensor lacked integration with microfluidics^33^. Mishra et al. developed an impedimetric CD4^+^ cell sensor that analyses the impedance changes when cells bind to its surface^35^. The selective capturing of CD4^+^ cells was achieved by functionalizing the electrode with anti-CD4 antibody. While the device was tested using peripheral blood mononuclear cells (PBMC) and lysed blood, it exhibited relatively high response variability (R^2^ = 0.49 for PBMC and R^2^ = 0.85 for lysed blood). Further characterization is needed to assess the linear range and the limit of detection. Additionally, the absence of integration with a microfluidic setup limits the applicability of the device. A sensor developed by Sher and Asghar is one of the closest designs to meet all key requirements for point-of-care use in low-resource settings, offering affordability and portability for rapid CD4^+^ cell quantification within the concentration range from 25 to 800 cells/µL^36^. The microfluidic platform combines an immunomagnetic separation site for capturing CD4^+^ cells with an impedimetric electrochemical sensor for enumeration, facilitating automated processing that minimizes manual handling. Although the authors used magnetic beads tagging for cell isolation, alternative approaches leveraging unique cell properties, such as size, shape, deformability, morphology, magnetic and electrical properties, compressibility, and specific surface markers, could eliminate the need for labeling.

This work aims to address the limitations of existing sensors by designing an easy-to-fabricate microfluidic platform for the electrochemical detection of CD4^+^ cells, which could be integrated with a cell-separation DFF chip to create a user-friendly, portable device for point-of-care applications (see Supplementary Fig. S1 in Supplementary Information). CD4^+^ cell detection was performed on the surface of a 3-mercaptopropionic acid self-assembled monolayer functionalized with anti-CD4 antibodies. The change in electrical properties at the electrode-electrolyte interface upon cell binding was recorded by electrochemical impedance spectroscopy (EIS). The sensor has been successfully applied to various biological samples, including recombinant human CD4 protein, Jurkat leukemic T cell lines, and primary CD4^+^ T cells isolated from blood. Unlike in other devices described in the literature, both sensor surface functionalization and cell detection are achieved within the microfluidic setup, reducing manual handling and increasing the repeatability of the process. The developed sensor has a broad linear detection range that spans both unhealthy and healthy levels of CD4^+^ cells in blood, which is critical for assessing the infection stage and monitoring treatment efficacy. The design also facilitates easy multiplexing for the detection of other clinically relevant markers. Additionally, the surface chemistry can be readily modified to capture other cells of interest by replacing the anti-CD4 antibody with an antibody for a target of interest. Finally, a previously developed inertial microfluidic DFF chip was employed to separate monocytes/neutrophils from PBMC (peripheral blood mononuclear cells), which were then used in the microfluidic device for interference tests. The CD4^+^ cell sensor was designed to be compatible with the DFF chip for future integration, which would eliminate the need for off-chip sample pretreatment. Preliminary data confirm the chip’s suitability for preconcentrating CD4^+^ cells for downstream detection. Overall, this work provides substantial foundations for the development of a fully integrated point-of-care HIV management device which could be used in low-resource settings as an alternative to the current laboratory-based gold standards. The developed sensor paired with the available engineering solutions is a scientific advancement towards the development of a novel device for accessible HIV management which aims to improve treatment adherence.

## Results

### Electrochemical characterization of the sensor

The sensor surface was characterized by performing faradaic EIS in the presence of a redox couple ([Fe(CN)_6_]^3-/4-^). The electrochemical properties of the sensor were modeled using the Randles circuit, and the charge transfer resistance after each step of the electrode functionalization was analyzed, for both static and flow measurements. As observed in Supplementary Fig. S2 (see Supplementary Information), a semicircle in the high-frequency region corresponds to the charge transfer resistance at the electrode/electrolyte interface. The formation of the self-assembled monolayer (SAM) leads to a repulsive interaction between the negatively charged redox probe, [Fe(CN)₆]³⁻/⁴⁻, and the negatively charged end groups of the SAM (–COO^-^), which acts as an insulating layer, resulting in an increase in charge transfer resistance. The resistance value further increases upon the addition of the antibody, primarily due to the hindrance effect that restricts the proximity of the redox probe to the electrode surface, thereby decreasing the electron transfer kinetics. Finally, the addition of blocking agents leads to a further increase in charge transfer resistance.

In order to ensure the proper development of a sensor module that can be integrated with microfluidic cell separation chips, four different biosensors were developed in this study: 1) sensors for purified CD4 protein, in static mode; 2) sensors for CD4^+^ cells from cell lines, in static mode; 3) sensors for CD4^+^ cells from cell lines, in flow mode; 4) sensors for CD4^+^ T-cells isolated from human blood samples, in flow mode.

**Fig. 1:**
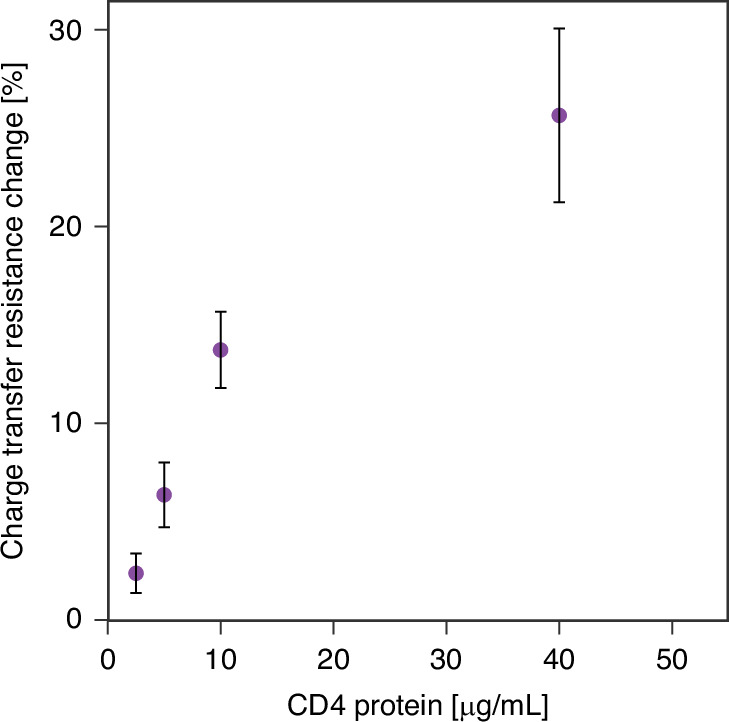
Charge transfer resistance changes upon the sensor’s incubation in increasing concentrations of CD4 protein. Faradaic EIS was performed in 0.01 M PBS + 0.1 M KCl in the presence of with applied DC potential of 0.2 V and AC potential of 0.01 V versus Ag/AgCl. The frequency was scanned from 10 kHz to 0.1 Hz. The error bars represent the standard deviation obtained from three independent replicates

As a preliminary study, the 10-electrode static setup was assessed using purified CD4 protein to confirm the specificity of the selected antibody as a biorecognition element. As presented in Fig. 1, the response was linear within the tested CD4 concentration range of 2.5–40 μg/mL. It is important to note that the detection of free CD4 protein is not clinically relevant, as CD4 is significant only as a membrane receptor on T cells. Thus, for further validation and characterization of the sensor, we used cells expressing the CD4 receptor on their surface to assess its performance under more physiologically relevant conditions.

**Fig. 2:**
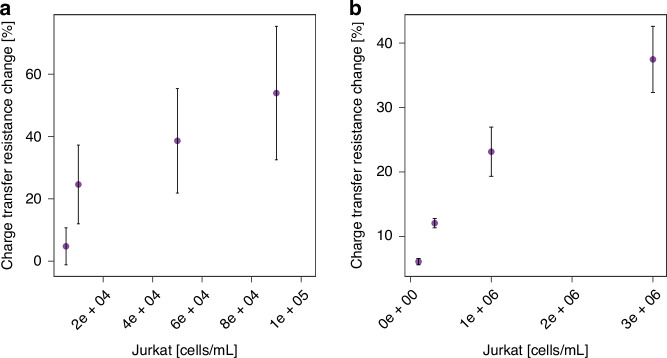
Sensor response for Jurkat cells. Calibration curve – percentage charge transfer resistance changes upon the sensor’s incubation in increasing concentrations of Jurkat cells using (**a**) static (**b**) microfluidic sensor. Faradaic EIS was performed in 0.01 M PBS + 0.1 M KCl in the presence of with applied (**a**) DC potential of 0.2 V and AC potential of 0.01 V versus Ag/AgCl (**b**) DC potential of 0 V and AC potential of 0.01 V vs Au pseudo-reference electrode. The frequency was scanned from 10 kHz to 0.1 Hz. The error bars represent the standard deviation obtained from three independent replicates

In the next step, the sensor was tested with a Jurkat cell line, an immortalized CD4^+^/CD8^-^ T lymphocyte cell line established in the mid-1970’s that was originally obtained from the peripheral blood of a boy with T cell leukemia that expresses an endogenous TCR (T cell receptor). The Jurkat cell line has been extensively used as a prototypical T cell line to study multiple events in T cell biology and HIV infection life cycle. The Jurkat cell model is a relatively cheap and easily replenishable source of lymphoid cells can simulate the function T lymphocytes, so it is widely used in the in vitro studies of T cell signal transduction, cytokines, and receptor expression, and can provide reference and guidance for the treatment of various infectious diseases and the research on their pathogenesis. Despite broad application of Jurkat cell line to study HIV infection life cycle, a few publications report inconsistent or low expression levels of this glycoprotein^37–39^. Hence, the expression of CD4 glycoprotein in our cells was confirmed with flow cytometry (See Supplementary Fig. S4, Supplementary Information). As seen in Fig. 2a, a concentration-dependent response to Jurkat cells was observed, but with high standard deviation reflected in large error bars. Key sources of variability, such as inconsistent incubation times, temperature fluctuations, and differences in antibody binding efficiency, are significant factors affecting the reproducibility and reliability of the static method. While efforts were made to maintain consistency during the experiments – such as electrode stabilization in PBS prior to measurements, standardizing incubation protocols and conducting procedures under controlled environmental conditions – certain factors, like the random orientation of antibodies on the sensor surface, remain inherently difficult to control. Manual pipetting can lead to non-uniform antibody coverage, directly impacting reproducibility and sensor performance. Additionally, reagents may dry unevenly during manual deposition, further contributing to variability.

**Fig. 3:**
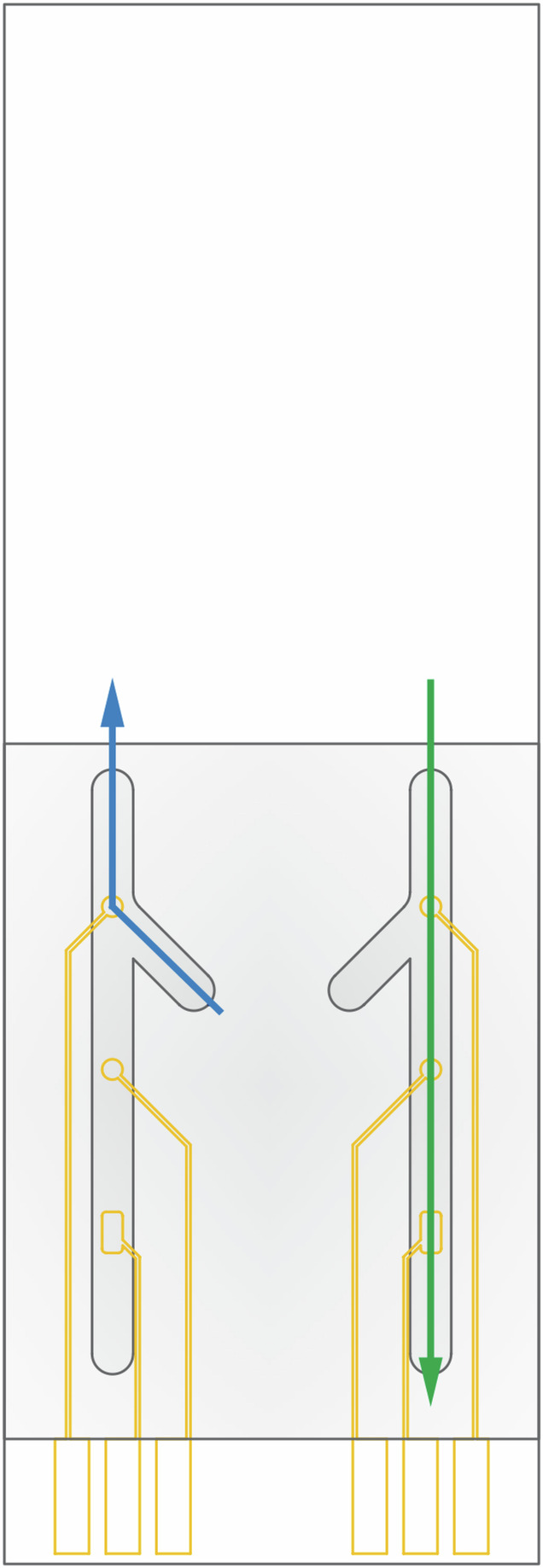
Microfluidic sensor design. Schematic of a microfluidic sensor consisting of a glass substrate with evaporated gold electrodes (2 sets that work as individual sensors) bonded with a PDMS chip (hatched). When the functionalization channel is used only the working electrode is exposed to the solution flowing in the direction indicated by the blue arrow. In contrast, using the main channel exposes all the electrodes to the solution flowing in the direction indicated by the green arrow. Thus, the main channel is only used for the measurements post-functionalization

The experiments with a static sensor demonstrated its possible suitability for the detection of CD4^+^ cells. However, it is not suitable for point-of-care applications due to the large reagent volumes required, the bulky setup with external working and counter electrodes, exposure to reagents, extensive manual handling, low measurement repeatability, and incompatibility with other microfluidic devices for sample processing. To advance the sensor toward its final application in low-resource settings by reducing manual handling and enabling compatibility with other microfluidic systems, such as those for cell separation, it was transitioned into a microfluidic platform. For this purpose, the sensor was redesigned, as illustrated in Fig. 3, to incorporate all electrodes (working, counter, and reference electrodes) onto a single chip, unlike the static setup, which relied on external reference and counter electrodes. With all electrodes made of gold, the fabrication process was straightforward and involved a single-step evaporation. Furthermore, the chip includes two sets of electrodes, enabling duplicate measurements or multiplexing with another sensor, thereby enhancing diagnostic capabilities. By utilizing the same electrode material and fabrication strategy as with the static setup previously described, one can evaluate the impact of microfluidics design on sensor performance while ensuring consistency in the electrode properties. This comparison is essential for validating the benefits of the microfluidic approach, particularly in improving measurement efficiency and accuracy.

Silicon, glass, and polymers are commonly used materials for microdevice fabrication, each offering distinct advantages: silicon for its thermal conductivity, glass for transparency, and polymers for cost-effectiveness and flexibility. Among these, PDMS (polydimethylsiloxane), which was utilized for the microfluidic channels, stands out due to its low manufacturing cost, flexibility, and suitability for medical applications. With an elasticity modulus of 1–3 MPa, PDMS closely resembles biological tissues, such as blood vessels. It is chemically inert, thermally stable, optically transparent, biocompatible, and gas-permeable, making it ideal for microfluidic and biosensing applications. PDMS is also easy to handle, capable of replicating submicron features to develop microstructures, and bonds well with glass substrates (taking advantage of the technological possibilities offered by glass substrates, such as metal deposition, oxide deposition, or surface functionalization, as demonstrated in the present work) or other PDMS layers via plasma treatment. Its deformability facilitates the integration of microfluidic valves, leak-proof fluidic connections, and the detection of low forces, such as biomechanical interactions from cells.

The microfluidic chip was designed to enable on-chip sensor functionalization and measurements. To functionalize the working electrode with an anti-CD4 antibody, various reagents must be injected into the channel. Using a single, long channel that exposes all three electrodes was not feasible, as it would lead to the functionalization of not only the working electrode but also the reference and counter electrodes. Thus, a dedicated functionalization channel was introduced, allowing reagents to flow exclusively over the working electrode. The channel was tilted to cross the main channel at a 45° angle to minimize the risk of fluid backflow into the main channel toward the reference electrode. The design was further simplified by using the main channel’s inlet as the outlet for the functionalization channel.

The microfluidic setup was first tested with Jurkat cells. Similarly to the static sensor, the microfluidic sensor showed a linear response to the increasing concentrations of Jurkat cells but exhibited significantly lower standard deviation (Fig. 2b). The microfluidic setup demonstrated a lower standard deviation compared to the static setup due to several key factors inherent in its design and operation. In the static sensor, each step of the electrode functionalization is performed manually, which introduces variability due to inconsistent distribution and deposition of reagents across the sensor surface. The static setup suffers from issues such as uneven mixing, slower diffusion rates, and larger volumes of reagents, all of which can introduce more variability. In contrast to a manual setup, the flow pump ensures controlled and uniform fluid flow, which reduces variability in the sample volume, reaction time, and reagent concentration. The precise channel dimensions and integrated flow control allow for more consistent interactions between the target analyte and the detection surface, enabling consistent coating and optimal antibody binding. It minimizes human errors in reagent volume, particularly at microliter scales, and provides precise reagent removal and surface washing. These factors collectively enhance the reproducibility of the sensor. This, together with minimized user exposure to reagents and tested samples, highlights the superiority of the microfluidic sensor over the static sensor.

**Fig. 4:**
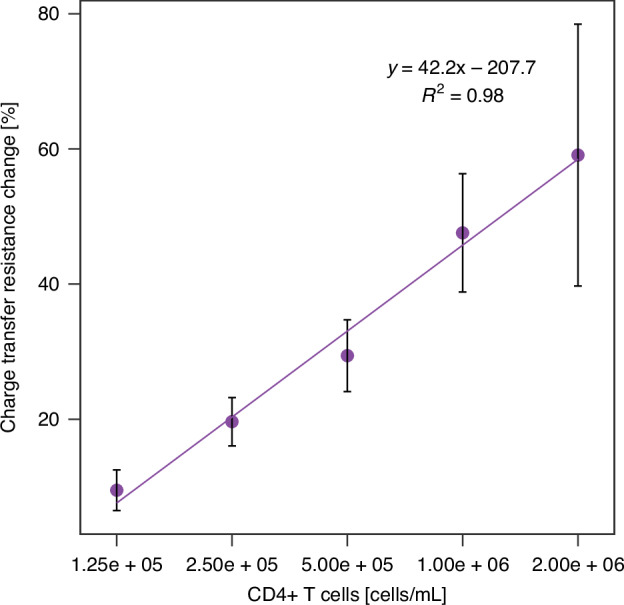
Sensor response to CD4^+^ T cells. Calibration curve – percentage charge transfer resistance changes upon the incubation of the microfluidic sensor with increasing concentrations of CD4^+^ T cells isolated from blood (suspended in 0.01 M PBS, pH 7.4 + 0.1 M KCl). Faradaic EIS was performed in 0.01 M PBS, pH 7.4 + 0.1 M KCl in the presence of 5 mM ferrocyanide and 5 mM ferricyanide with applied DC potential of 0 V and AC potential of 0.01 V vs Au pseudo-reference electrode. The frequency was scanned from 10 kHz to 0.1 Hz. The error bars represent the standard deviation obtained from three independent replicates

Finally, the applicability of the microfluidic sensor for the final application was assessed by monitoring its response to increasing concentrations of freshly isolated CD4^+^ cells from blood (Fig. 4). The response was concentration-dependent and linear within the range of 1.25 × 10^5^ to 2 × 10^6^ cells/mL. The sensor responses across different concentrations were analyzed using One-Way ANOVA, revealing a statistically significant difference between groups (*p*-value of 0.000724). This indicates that the changes in sensor response are significant, supporting the reliability of the observed trends in the calibration curve.

The limit of blank (LOB) represents the highest analyte concentration that can be attributed to the background response of a sample containing no analyte, whereas the limit of detection (LOD) represents the lowest analyte concentration that can be reliably distinguished from the LOB. The LOB and LOD of the sensor were calculated as 1.03 × 10^5^ cells/mL and 1.41 × 10^5^ cells/mL, respectively, as follows^40^:

$${\rm{LOB}}={{\rm{mean}}}_{{\rm{blank}}}+1.645\times {{\rm{SD}}}_{{\rm{blank}}}$$

$${\rm{LOD}}={\rm{LOB}}+1.645\times {{\rm{SD}}}_{{\rm{low\; concentration\; sample}}}$$

The developed sensor demonstrates a linear range that effectively encompasses both healthy (1.5 × 10^6^ – 5 × 10^5^ cells/mL) and unhealthy (<5 × 10^5^ cells/mL) levels of CD4^+^ cells in blood, making it highly suitable for assessing the stage of infection. Although other sensors reported in Table 1 have lower detection limits extending to significantly lower CD4^+^ cell levels, this capability is not advantageous unless the sample is diluted. However, sample dilution is ideally avoided in clinical practice to minimize processing steps. The linear range and limit of detection of the developed sensor is sufficient to detect even critically low CD4^+^ cell levels indicative of AIDS (<2 × 10^5^ cells/mL), ensuring reliable detection without the need for dilution. Furthermore, the upper limit of the linear range for this sensor is the highest among the reported devices. This extended range enables monitoring immune recovery following therapy initiation and detecting early signs of immune system deterioration before the onset of clinical symptoms.

**Table 1:** Comparison of the electrochemical CD4^+^ cell sensors described in the literature

Sample	Target cells capture	Detection	Mechanism	Comments	Linear range [cells/mL]	LOD [cells/mL]	Reference
Whole blood	Immuno-magnetic separation	Amperometry	HRP as an electrochemical reporter	T cell separation by two-step labeling of the CD3 and CD4 markers. 3-electrode setup, not integrated into a miniaturized device.	8.9 ×10^4^ – 9.12 ×10^5^	4.4 ×10^4^	Carinelli et al.^34^
Heterogenous cell suspension	Antibody-functionalized electrode	SWV		High sensitivity (4.55 × 10^−2^ μA) and low manufacturing cost. Long fabrication time (4 – 5 weeks). Not integrated with microfluidics.	1 ×10^2^ – 1 ×10^6^	1 ×10^2^	Kim et al.^33^
Lysed whole blood	Antibody-functionalized electrode	EIS	Changes in the electrical properties of the system upon the cell binding to the electrode	Protein G for antibody functionalization via the Fc region	N/A	N/A	Mishra et al.^35^
Whole blood	Immuno-magnetic separation	Electrical impedance		Disposable, low sample volume, microfluidic setup	2.5 ×10^4^ – 8 ×10^5^	2.5 ×10^4^	Sher and Asghar^36^
Blood-isolated CD4^+^ cell suspension	Antibody-functionalized electrode	EIS		Microfluidic setup, compatible with DFF device, suitable for multiplexing	1.25 ×10^5^ – 2 ×10^6^	1.41 ×10^5^	This work

### Specificity study

**Fig. 5:**
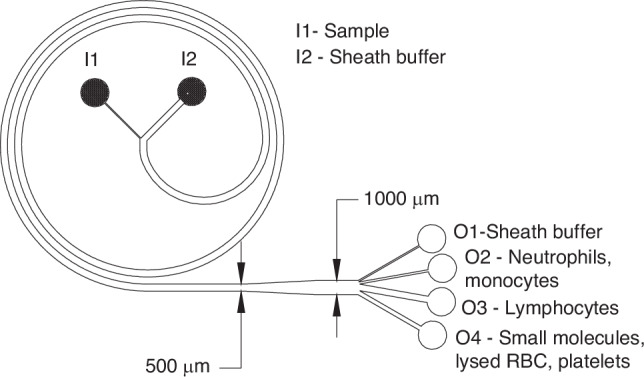
A schematic illustration of the DFF chip used for cell separation. I1 and I2 indicate inlets for sample and sheath buffer, respectively. O1 – O4 indicate outlets for collecting particles of varying sizes, enabling the separation of cells of interest. Further details on the chip design and functionality can be found in Hou et al.^41^

Specificity is a critical factor for real-world applications of sensors, as nonspecific interactions can compromise detection accuracy, leading to misdiagnosis and suboptimal treatment, thereby jeopardizing patient health and safety. Obtaining specificity can be especially challenging when detection is performed in complex body fluids, which are rich in nontarget molecules. Thus, the sensor was designed with the intention of integrating it with a label-free, high-throughput microfluidic DFF chip for cell separation shown in Fig. 5^41,42^. This 2-inlet, 4-outlet sorting device utilizes a spiral geometry in which cells are subjected to inertial microfluidics, a passive particle separation method. In this approach, particles’ positions within the channel cross-section are governed by the equilibrium between two counteracting forces: wall lift force and shear lift force, which together constitute the total lift force (F_L_). Additionally, in the spiral channel of the DFF chip, a secondary flow, called Dean flow, arises due to centrifugal fluid acceleration. As a result, particles experience Dean drag forces (F_D_), which move them along the Dean vortices. The interplay between the lift and Dean drag forces determines the particles’ equilibrium position within a channel cross section enabling the precise separation of cells based on their size.

**Fig. 6:**
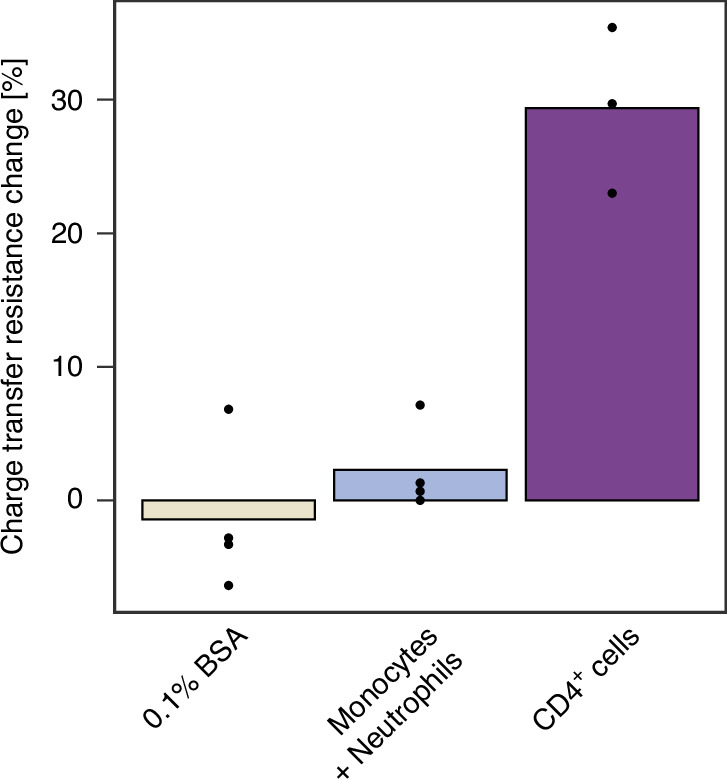
Specificity of the sensor. Sensor’s response to 0.1% BSA, neutrophils + monocytes (isolated with the DFF-chip), and CD4^+^ cells (isolated from blood using EasySep™ Human CD4^+^ T Cell Isolation Kit). All solutions were prepared in 0.01 M PBS, pH 7.4 + 0.1 M KCl + 0.1% BSA at the concentration of 0.5 × 10^6^ cells/mL. Black points represent triplicates of each measurement

0.1% BSA (in PBS) is a commonly used sheath buffer in cell sorting, including the described DFF chip, and it is essential to ensure that it does not cause nonspecific binding. Additionally, BSA shares a high degree of similarity with HSA (human serum albumin) present in blood, which can potentially interfere with target detection. Therefore, the sensor was tested with a 0.1% BSA solution. Additionally, the sensor was tested with a mixture of monocytes and neutrophils (0.5 × 10^6^ cells/mL) isolated from PBMCs using the DFF chip to further verify its specificity. Both 0.1% BSA and non-lymphocytes caused a negligible change in charge transfer resistance (Fig. 6), providing direct evidence of the sensor’s specificity towards CD4^+^ cells. The sensor responses were analyzed using One-Way ANOVA, revealing a significantly higher response to CD4^+^ cells compared to potential interferents (0.1% BSA and a mixture of monocytes and neutrophils) (*p*-value of 0.000925). This supports the specificity of the CD4^+^ cell sensor for its intended application. Nevertheless, the sensor’s selectivity should be further investigated to confirm its ability to accurately detect CD4^+^ cells in the presence of non-target cells and proteins found in complex body fluids such as blood.

## Discussion

Polymerase chain reaction (PCR) and loop-mediated isothermal amplification (LAMP) are well-established methods for the accurate detection of HIV by amplifying and identifying viral genetic material. While PCR and LAMP are highly sensitive and specific, they require complex instrumentation and skilled operators, limiting their portability and suitability for point-of-care applications. These methods also involve time-consuming amplification steps and extensive sample preparation, making them expensive and less accessible in resource-limited settings. Alongside these genetic approaches, several non-genetic tests, such as antibody and antigen detection assays, are commonly used in low-resource settings for diagnosing HIV. While monitoring viral load is a critical parameter in managing HIV, the CD4^+^ T cell count is equally valuable as it provides essential information about the state of the immune system, the progression of the disease, and the effectiveness of antiretroviral therapy. Together, these metrics are fundamental for comprehensive patient care.

Existing methods for measuring CD4^+^ T cell counts, such as flow cytometry, are not well-suited for point-of-care testing in low-resource settings. They are expensive, require skilled operators, and depend on sophisticated laboratory infrastructure, limiting their accessibility in regions where they are most needed. Thus, electrochemical sensors offer significant promise due to their affordability, portability, and rapid response times. Unlike optical methods, electrochemical sensors can be easily integrated into compact, low-cost devices, enabling reliable CD4^+^ T cell counting at the point of care. Their minimal sample preparation requirements, ease of use, and ability to operate on battery power make them ideal for deployment in remote and resource-constrained environments. By leveraging the advantages of electrochemical sensors, we provide a solution that helps to bridge the gap in HIV care by making CD4^+^ T cell monitoring accessible, scalable, and cost-effective, thereby improving patient outcomes, and supporting the global fight against HIV.

As demonstrated in Table 1, this work achieves results comparable to those reported in the literature while potentially introducing a lab-on-chip system that integrates cell separation with electrochemical detection within a microfluidic design. This innovative approach enhances operational efficiency, ensures ease of use in resource-limited settings, and holds significant potential for advancing point-of-care applications. The reported CD4^+^ cell sensor is one of the two EIS-based devices and the only one integrated with microfluidic system. The sensor has demonstrated responsiveness to both CD4 protein and CD4^+^ cells, including the Jurkat cell line and CD4^+^ cells isolated from blood. Although the LOD is not as low as that of other sensors, it is sufficient to detect even critically low levels of CD4^+^ cells in the blood of patients who have developed AIDS. The sensor’s linear response to the blood-isolated CD4^+^ cells (1.25 × 10^5^ – 2 × 10^6^ cells/mL) encompasses both unhealthy and healthy CD4^+^ cell levels, demonstrating its suitability for assessing the prognosis of HIV^+^ patients. The extended upper range, compared to other sensors, enables the monitoring of even minor immune system deterioration before the onset of symptoms, which is crucial for early diagnosis. To the best of our knowledge, the sensor developed in this work is the only one that enables both functionalization and measurement within a single setup, thanks to the dual operation modes of the microfluidic chip. This design shortens the procedure, minimizes human input, and automates the process using syringe pumps, thereby enhancing detection reproducibility^43^. The sensor’s specificity was demonstrated by its negligible response to BSA and a monocyte/neutrophil cell suspension (0.5 × 10⁶ cells/mL) derived from PBMCs using the Dean flow fractionation microfluidic chip. Although the sensor was not tested in whole blood as some of the sensors described in the literature (Table 1), the use of an inertial microfluidic chip for cell separation can reduce potential interference arising from complex samples; however, further optimization and clinical testing are necessary.

**Fig. 7:**
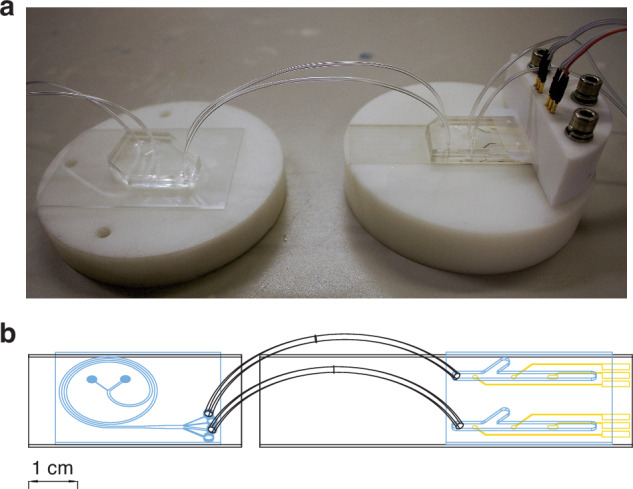
Integration of the sensor with the DFF chip. An image (**a**) and a simplified schematic (**b**) of the integrated DFF chip (left) with the CD4^+^ cell sensor (right). PDMS chips are shown in blue, and gold electrodes in yellow. Tubing was indicated only for the connection of the DFF chip with the sensors. Tubing for waste and the holders are not shown in the schematic for the sake of clarity. Device operation: The tested sample is first introduced to the DFF chip, where it is separated into fractions based on particle size. The fraction containing lymphocytes is then directed to the CD4^+^ cell sensor, which captures CD4^+^ cells and enumerates them by measuring the change in impedance at the electrode/electrolyte interface

The sensor is suitable for integration with a DFF chip^43^, capable of an on-chip sample preprocessing for downstream CD4^+^ cell detection. Figure 7 presents a simplified diagram of the components required for a modular device for HIV management which allows for multiplexing CD4^+^ sensor. A preliminary study using flow cytometry confirmed that the chip is suitable for lymphocyte separation and preconcentration for downstream CD4^+^ cell detection (see Supplementary Fig. S3, Supplementary Information). The chip can be modularly coupled with or seamlessly integrated with the CD4^+^ cell biosensor, moving towards a point-of-care lab-on-chip device. The integration of DFF into the microfluidic device simplifies the cell separation process by automating what are traditionally time-consuming and labor-intensive steps, such as centrifugation and filtration. This allows the device to directly process serum samples, efficiently separating target cells using distinct channel properties based on cell size, surface properties, and density. The target cells are directed toward the detection zone, where they are captured by functionalized working electrodes, ensuring selective detection while non-target cells and debris are washed away. By automating the entire process, the system reduces human error, minimizes sample handling, and improves consistency and reproducibility. The device’s operation, controlled by running time and fluid dynamics, eliminates the need for skilled operators and manual intervention, making it ideal for point-of-care applications. Overall, DFF integration enhances efficiency and accessibility, especially in resource-limited settings, by streamlining sample processing and reducing the need for specialized training.

The proposed strategy shows promise in providing accessible HIV-monitoring tools to individuals, regardless of their geographic location, by translating conventional laboratory-based examination methods into a point-of-care device that can be delivered directly to patients. Future work should include optimizing the sensor’s integration with the DFF chip, conducting reproducibility studies, and testing with clinical samples. Furthermore, readily available engineering solutions should be explored to facilitate the sensor’s translation into a self-contained, low-footprint instrument.

## Materials and methods

### Chemicals

3-Mercaptopropionic acid (MPA), N-(3-Dimethylaminopropyl)-N′-ethylcarbodiimide hydrochloride (EDC), ethanolamine (EA), phosphate buffered saline tablets (PBS), RPMI-1640 medium (R8758), sodium pyruvate solution, N-(2-Hydroxyethyl)piperazine-N′-(2-ethanesulfonic acid) (HEPES) solution, penicillin-streptomycin, fetal bovine albumin, potassium chloride, Bright-Line™ hemacytometer, trichloro(1H,1H,2H,2H-perfluorooctyl) silane and Dulbecco’s phosphate buffered saline were purchased from Sigma-Aldrich. N-Hydroxysuccinimide (NHS), StartingBlock™ (PBS) Blocking Buffer, and Ethylenediaminetetraacetic acid (EDTA) were purchased from Thermo Fisher Scientific. Other reagents included EasySep™ Human CD4^+^ T Cell Isolation Kit (Stemcell Technologies), recombinant human CD4 protein (Abcam, ab245956), 2-(N-morpholino)ethanesulfonic acid (MES) Free Acid (EMD Millipore Corp.), microscope slides 76 × 26 mm (Academy), gold pellets (Testbourne Ltd), trypan blue stain 0.4% (Life Technologies), monoclonal anti-CD4 antibody (BioLegend), Jurkat cells (clone E6-1, ATCC, TIB-152™), recombinant human CD4 protein (Abcam), Ficoll-Paque™ PLUS (GE Healthcare), MACS® BSA stock solution (Miltenyi Biotec, 140-001-130.04), FITC anti-CD4 antibody (Abcam), PE/Cyanine 7 anti-CD14 antibody (301814, BioLegend), Human TruStain FcX™ (Fc Receptor Blocking Solution, BioLegend), potassium chloride (Sigma-Aldrich), and absolute ethanol (Sigma-Aldrich), Ficoll-Paque™ PLUS (GE Healthcare), MACS® BSA stock solution (Miltenyi Biotec).

### Sensor design and microfabrication

The electrode design was created in Autodesk AutoCAD considering the 76 × 26 mm glass slide as the electrode substrate. Electrodes were fabricated using metal thermal evaporation. A 10 nm layer of chromium was evaporated onto a clean glass slide, followed by an evaporation of 100 nm layer of gold. Chromium was used as an adhesion layer due to its strong bonding properties, whereas gold was used for electrode fabrication due to its high conductivity and ease of further functionalization with a self-assembled monolayer via strong gold-thiol bonding. Electrodes were patterned using an in-house-designed shadow mask.

For preliminary studies, a static set-up with a 10-electrode linear array was fabricated. Each of the 10 gold electrodes was an individual working electrode to which a counter electrode (platinum wire) and reference electrode (Ag/AgCl) were supplemented externally. Fitting 10 electrodes on a single substrate provided a practical advantage, as it allowed for testing multiple conditions in a single experiment, increased the number of experimental repeats, and improved efficiency by reducing the need for additional substrates.

**Fig. 8:**
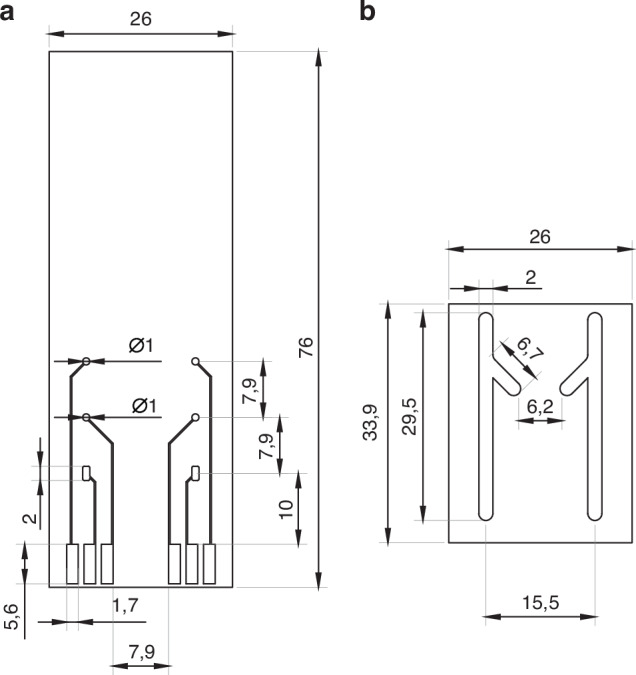
Schematic design of the sensor. The sensor consists of gold electrodes evaporated on a glass substrate (**a**) and the PDMS microfluidic chip (**b**). The sensor and the PDMS chip were then integrated to enable pump-controlled electrode functionalization and measurements. Dimensions are reported in millimeters

A final sensor design compatible with a microfluidic chip is presented in Fig. 8. This set-up included two sets of individual sensors, each consisting of three electrodes in the following order (from the top of the schematic): working electrode, reference electrode and counter electrode. Integration of the electrodes onto a single substrate enables the device miniaturization. The working and reference electrodes were 1 mm in diameter, whereas the area of the counter electrode was bigger (~2 mm^2^) to provide sufficient electron flow. The area of the counter electrode was larger than that of the working electrode to maintain low-resistance, stable, and unconstrained current flow.

### Microfluidic chip design and microfabrication

To automate the fabrication and detection processes, minimize manual sample handling, reduce sample volume, and integrate the sensor with the cell separation DFF chip, the sensor was incorporated into a microfluidic setup. The PDMS chip was designed in Autodesk AutoCAD as presented in Fig. 7b. The design of the microfluidic sensor is unique due to the two intersecting channels, which can be used independently. The main (long) channel was used for electrochemical measurements, whereas the side channel was used for functionalization of the working electrode and incubation with tested samples. Microfluidic channels were prepared by casting polydimethylsiloxane (PDMS) onto a previously fabricated master mold. The master mold was 3D-printed using clear resin v4 from Formlabs (RS-F2-GPCL, United States) and thoroughly washed with isopropanol following a 5-min sonication in isopropanol. The mold was then attached to a 100 mm Petri dish using double-sided tape and exposed to UV at 65 °C for 2 h, followed by baking at 75 °C for 24 h. Finally, the mold was exposed to silane vapor under a vacuum for an hour.

To prepare a device, PDMS was cast onto the master mold using a silicone elastomer base and curing agent (Sylgard™) mixed in a 10:1 ratio. The cast PDMS was left in a desiccator for 30 min to remove air bubbles, following baking at 75 °C for 1 h. After cooling down, the PDMS chip was cut out from the mold. Inlets and outlets were made with a 1.5 mm diameter hole punch. A PDMS chip and a substrate (glass slide with evaporated electrodes) were cleaned with isopropanol, and dried with nitrogen before both were placed in a plasma chamber. After complete air removal from the chamber by a vacuum pump, a carrier gas is introduced into the chamber, raising the chamber pressure to 1 mbar, exposing the samples to plasma for 1 min, at 100 W and immediately aligned to allow the bonding of the PDMS to the glass substrate. Finally, the device was baked at 75 °C for 1 h.

### Sensor surface functionalization

In short, the sensor surface was prepared in a multi-step process involving functionalization with a self-assembled monolayer, activation with EDC/NHS, attachment of a biorecognition element, and blocking the surface to minimize non-specific binding. Each step was designed to improve sensor specificity and stability.

All incubations were performed at room temperature. A working electrode was incubated for 20 h in 20 mM 3-mercaptopropionic acid (MPA) solution prepared in PBS (0.01 M, pH 7.4) following 2 h incubation in PBS. Next, the electrode was incubated for 30 min in a 1:1 mixture of 200 mM EDC and 50 mM NHS freshly prepared in MES (1 mM, pH 4.7), following the rinsing with MES and then with PBS. The electrode was then incubated for 30 min in a 10 µg/mL solution of anti-CD4 antibody and rinsed with PBS. The electrode was blocked first with ethanolamine (1 mM, pH 8.5) for 15 min and next with StartingBlock™ Blocking Buffer for 30 min. Finally, the electrode was stabilized in 0.01 M PBS for 20 h at room temperature.

For static sensor fabrication with no microfluidics integrated, reagents were simply pipetted in and out of a well exposing a working electrode. In the case of the microfluidic sensor, the human input was minimized by infusing channels with a syringe pump (kdScientific, USA), 2 mL plastic syringes and tubing (Saint Gobain, Tygon® Tubing) with an inner diameter of 0.02 inches. During the functionalization, the outlet of the main (long) channel was air-tight closed with tape to stop reagents from flowing towards the reference and counter electrode. To functionalize a working electrode, a reagent was injected through a functionalization (side) channel for 2 min at 50 µL/min. Next, the reagent was flushed away with PBS for 2 min before introducing the next reagent.

### Electrochemical measurements

An electrochemical cell was assembled using an in-house-fabricated Teflon holder with pogo pins for connecting the electrodes to a potentiostat. Electrochemical measurements were performed using a PalmSens4 potentiostat connected to a computer with PSTrace 5.7 software (PalmSens, The Netherlands). Faradaic EIS was performed with applied DC potential of 0.2 V and AC potential of 0.01 V *versus* an Ag/AgCl reference electrode. A gold pseudo-reference electrode in the microfluidic set-up was validated versus an external Ag/AgCl reference electrode by running cyclic voltammetry in 5 mM [Fe(CN)_6_]^3-^ and 5 mM [Fe(CN)_6_]^4-^ in PBS supplemented with 0.1 M KCl (0 V versus pseudo-reference electrode equals 0.2 V versus Ag/AgCl reference electrode). Randles circuit was then used to characterize the electrochemical circuit and extrapolate charge transfer resistance (R_CT_). The resistance change (ΔR_CT_) was then calculated as: (R_ct(sample)_ - R_ct(0)_) in which R_CT(0)_ is the signal of the blank sample and R_CT(sample)_ is the signal of the sample. The baseline (R_CT(0)_) was subtracted to account for the differences in the base resistance of replicates (electrode-to-electrode variations). All the measurements were recorded in triplicate and recorded as a means.

### Sensor characterization and CD4 protein/CD4^+^ cell detection

For CD4 protein or CD4^+^ cell detection using the static setup, an electrode was incubated in a protein/cell suspension for 30 min at room temperature and then washed with PBS. The incubation time was not optimized, but it was chosen based on the protocols found in the literature.

In the microfluidic device, the sample was flowed through a functionalization side channel for 2 min at a rate of 50 µL/min to ensure complete coverage of the electrode and to fully fill the microfluidic channels. The sample was then incubated in static mode for 30 min. Following incubation, the sample was removed from the channel by flushing it with PBS for 2 min to ensure complete clearance. Next, the inlet of the functionalization channel was air-tight closed, and the outlet of the main channel was opened to allow flow through the main channel as indicated in Fig. 3 (blue arrow). To ensure that no cells were left in the device, the main channel was flushed with PBS for another 5 min before applying the faradaic measurement.

### Cell culture

Jurkat cell line was cultured in suspension in a humidified atmosphere at 37 °C and 5% CO_2_. Growing medium RPMI-1640 was supplemented with 10% Fetal Bovine Albumin (FBS), 1% Penicillin-Streptomycin (100 I.U./mL Penicillin, 100 µG/mL Streptomycin), 1 mM sodium pyruvate and 10 mM HEPES. Before using the cells for measurements, they were counted using a hemocytometer and washed twice with PBS (0.01 M, pH 7.4).

### CD4^+^ cell isolation

Blood samples were collected from healthy donors, adhering to the ethical approval under the umbrella of the University of Bath, UK (Department for Health) and Nanyang Technological University, Singapore. Human peripheral blood mononuclear cells (PBMC) were isolated from a blood sample. First, a blood sample was mixed with 0.1% bovine serum albumin (BSA) in PBS (0.01 M, pH 7.4) in a 1:1 ratio. The mixture was layered on top of the Ficoll density-gradient reagent (a volume ratio of 1:1) and centrifuged for 30 min at 23 °C, 300 ×*g* (acceleration 7, deceleration 3). After the centrifugation, the sample was separated into 4 layers. The layer containing PBMC was removed and washed with 0.1% BSA (a volume ratio of 1:2). Centrifugation was set for 5 min at room temperature, 1300 rpm (acceleration 9, deceleration 9). The supernatant was discarded, and the pellet was resuspended in 0.25–1 mL of 1 mM EDTA with 2% FBS in PBS. Cells were counted and further processed to isolate CD4^+^ cells using EasySerp™ Human CD4^+^ T Cell Isolation Kit. The supplier’s protocol was followed. The isolated CD4^+^ cells were resuspended in 500 µL of 0.1% BSA and counted (10× dilution).

### DFF-chip monocytes/neutrophils isolation

A fresh blood sample was collected and processed on the same day. First, peripheral blood mononuclear cells (PBMC) were isolated by density gradient centrifugation as described above and suspended at the concentration 5–8 ×10^6^ in 0.1% BSA in 0.01 M PBS, pH 7.4 + 0.1 M KCl. For the size-based cell separation, a 2-inlet, 4-outlet Dean Flow Fraction (DFF) chip developed at the Nanyang Technological University in Singapore was used (Fig. 5)^41,42^. First, a DFF chip was primed with 70% ethanol to disinfect the channels and minimize occurrence of bubbles. Next, the sheath and sample inlets were connected to a 50 mL syringe containing sheath buffer (0.1% BSA in 0.01 M PBS) and a 3 mL syringe containing PBMC suspension, respectively. The syringes were secured to a syringe pump and perfused at an optimized flow speed of 1300 µL/min for sheath and 130 µL/min for isolation of monocytes/neutrophils^42–44^. After stabilizing the flow for 2 min, separated monocytes/neutrophils were collected at the outlet O2 as depicted in Fig. 5. Collected cells were centrifuged and re-suspended in 0.01 M PBS, pH 7.4 + 0.1 M KCl for specificity study.

## Supplementary information

Supplementary Information
