# Supplementary material for: Electrochemical microfluidic biosensor for the detection of CD4+ T cells
Source: Microsyst Nanoeng. 2025 Apr 9;11:63. doi: 10.1038/s41378-025-00893-8 (PMC11982565; doi:10.1038/s41378-025-00893-8)
Supplement: Supplementary file 1 — Supplementary Information [file 41378_2025_893_MOESM1_ESM.docx]

**Electrochemical Microfluidic Biosensor for the Detection of CD4^+^ T-Cells**

Katarzyna Białas, Hui Min Tay, Chayakorn Petchakup, Razieh Salimian, Stephen G. Ward, Mark A. Lindsay, Han Wei Hou, Pedro Estrela

**Supplementary Information**

Idealized device

Figure S1 depicts a schematic of a full device for CD^+^ T-cell quantification, comprising of a DFF chip for lymphocyte separation and a CD4^+^ cell sensor module.


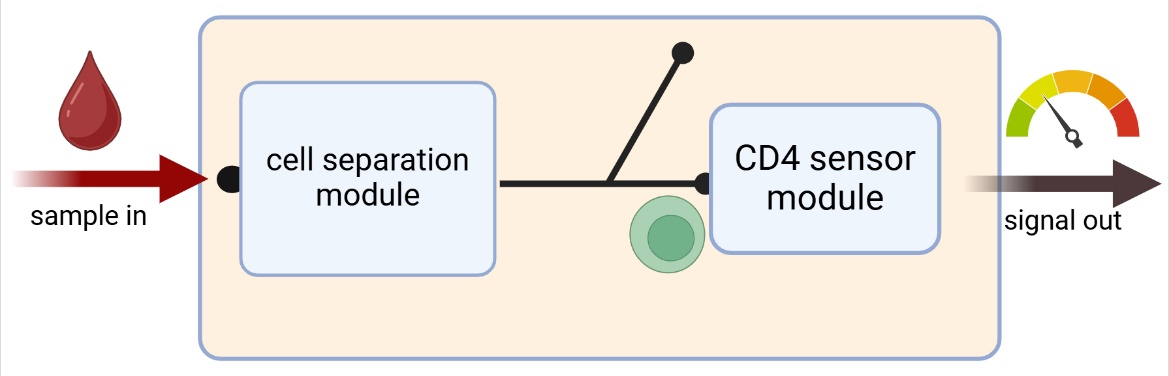


*Figure S1. Schematic of a device integrating a cell separation module (DFF chip) and a CD4^+^ cell sensor module.*

Impedance measurements

The sensor surface was characterized by performing Faradaic electrochemical impedance spectroscopy in the presence of a redox couple (ferro/ferricyanide). The electrochemical properties of the senor were modelled using the Randles circuit, and the charge transfer resistance after each step of the electrode functionalization was analyzed and reported in Table S1. The charge transfer resistance of a bare gold electrode (GE) increased upon self-assembled monolayer formation (SAM/GE). A further increase was observed after anti-CD4 antibody attachment to SAM (Ab/SAM/GE), and finally, after blocking with ethanolamine and StartingBlock™ (PBS) Blocking Buffer (Ab/SAM/GE *blocked). Every step of the functionalization process resulted in an increased charge transfer resistance by creating a physical barrier for electron flow. The same tendency was observed for a static and microfluidic setup (Figure S2).

*
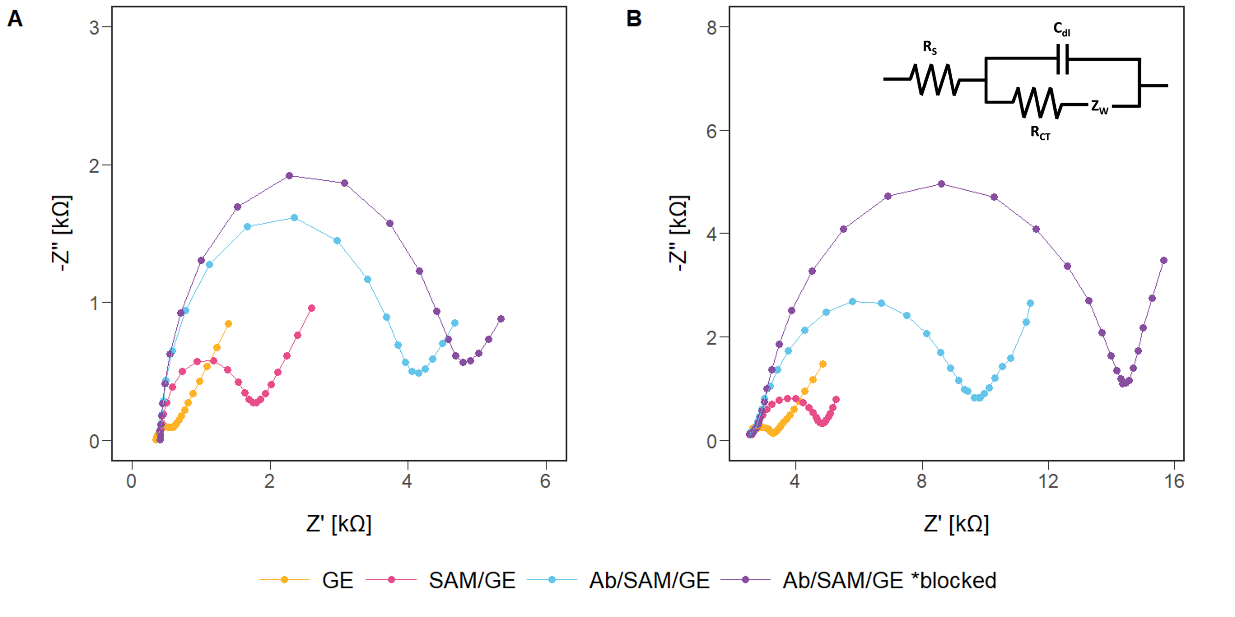
*

*Figure S2. (A) Static and (B) microfluidic sensor surface characterization – Nyquist plots representing various stages of sensor fabrication. Faradaic EIS was performed in 0.01 M PBS, pH 7.4 + 0.1 M KCl in the presence of 5 mM [Fe(CN)_6_]^3-/4-^ with applied AC potential of 0.01 V and DC potential of (A) 0.2 V vs Ag/AgCl (B) 0 V vs Au pseudo-reference electrode. The frequency was scanned from 10 kHz to 0.1 Hz. The inset in figure B shows the Randles circuit: R_S_ – solution resistance, R_CT_ – charge transfer resistance, C_DL_ – double layer capacitance, Z_W_ – Warburg impedance.*

*Table S1. Charge transfer resistance changes upon each step of electrode surface modification in the static and flow microfluidic setup.*

| Electrode | Static | Flow |
| --- | --- | --- |
| GE | 191 Ω | 654 Ω |
| SAM/GE | 990 Ω (+ 418.3%) | 1863 Ω (+ 184.9%) |
| Ab/SAM/GE | 3179 Ω (+ 221.1 %) | 6088 Ω (+ 226.8%) |
| Ab/SAM/GE *blocked | 3708 Ω (+16.6 %) | 10420 Ω (+ 71.2%) |

Flow cytometry

Figure S3 shows a density dot plot that allows for a two-parameter analysis and identification of single positive (for each parameter) cells as well as double positive and double negative cells. As expected, there were distinct populations of both monocytes and CD4^+^ lymphocytes in the PBMC sample. However, the sample collected at outlet O3 was depleted of monocytes, which confirms that they were separated from lymphocytes due to the size difference between these cell types of PBMC. The monocyte content in the sample before the on-chip DFF is 17.73% and it drops to 0.82% in the sample collected at outlet O3 after the separation. On the other hand, the CD4^+^ lymphocytes content increases from 33.72% before the separation to 48.48% after the separation, confirming the DFF microfluidic chip suitability for the lymphocyte separation from other cells subpopulations (such as monocytes) and preconcentration for the downstream CD4^+^ cells detection. This experiment confirmed that the DFF microfluidic chip is suitable for lymphocyte separation and preconcentration for downstream CD4^+^ cell detection.


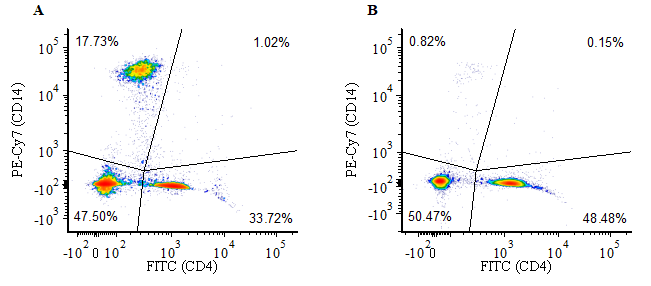


Figure S3. Dot plots illustrating the changes in the cell subpopulations (A) before and (B) after the on-chip DFF. CD4 and CD14 were used as markers of T-cells and monocytes, respectively. Anti-CD4 and anti-CD4 antibodies coupled with FITC or PE-Cy7, respectively, were used to allow flow cytometry-based cells detection. Quadrant gate consists of a double negative population in the bottom left corner (CD4^-^ and CD14^-^ cells), a double positive population in the upper right corner (CD4^+^ and CD14^+^ cells), a single, FITC, positive subpopulation in the bottom right corner (CD4^+^ cells), and a single. PE-Cy7, positive subpopulation in the upper left corner (CD14^+^ cells). Gate: singlets, number of events: 16,469.

CD4 expression on Jurkat cells

The CD4 expression on the surface of Jurkat cells was assessed by flow cytometry. MCF7, a breast cancer cell line that does not express CD4, was used as a negative control. FITC-labeled anti-CD4 monoclonal antibody (ab59474, Abcam) was used for staining. As shown in Figure S4, the FITC signal for Jurkat cells reached saturation at an antibody concentration of 0.2 µg per 1 × 10^6^ cells. Approximately 80% of Jurkat cells were CD4-positive, confirming the suitability of this cell line for use in the CD4^+^ cell sensor study. As expected, the MCF7 cell line showed minimal binding of the anti-CD4 antibody due to the lack of CD4 expression. The percentage of positive MCF7 cells was below 1%, which is negligible and likely due to very low background staining.


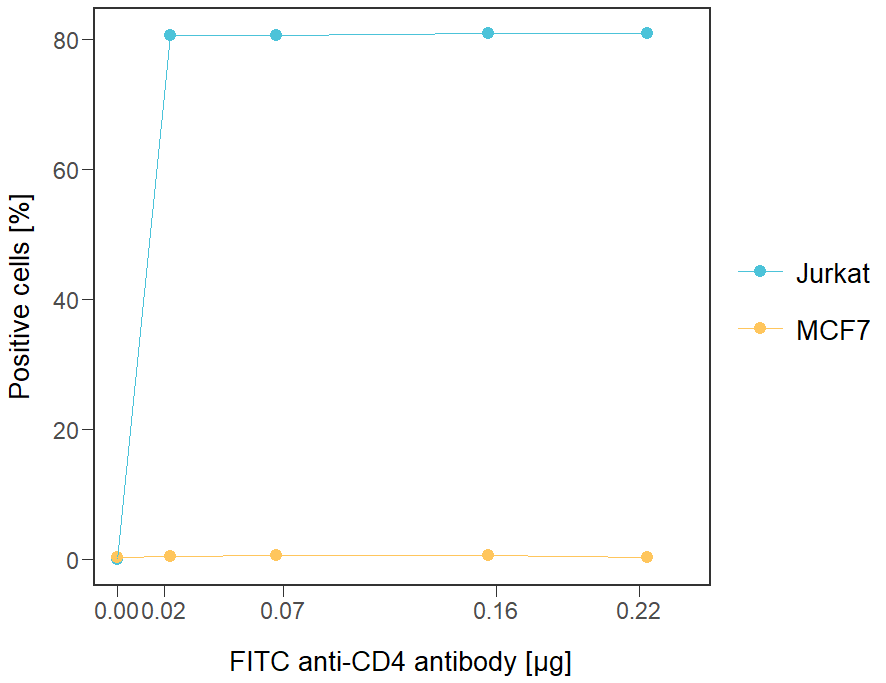


Figure S4. Flow cytometry analysis of CD4 expression by different cell lines. Cells were stained with increasing concentrations of FITC anti-CD4 antibody. Voltage set to 320 V. 20,000 events were recorded for each sample.
